# Supplementary material for: Correction of the Splicing Defect Caused by a Recurrent Variant in ABCA4 (c.769-784C>T) That Underlies Stargardt Disease
Source: Cells. 2022 Dec 7;11(24):3947. doi: 10.3390/cells11243947 (PMC9777113; doi:10.3390/cells11243947)
Supplement: Supplementary file 1 [file cells-11-03947-s001.zip › cells-2028742-supplementary.pdf]

Supplementary Figure S1. Sequence analysis of the resulting RT-PCR products from rescue experiments of the deep-intronic variants c.769-784C>T causing insertion of pseudoexon. Sanger sequencing chromatograms from splicing correction experiments targeting c.769-784C>T. **A)** Sequencing results in HEK293T-midigene system: sequences of the correct transcript from wild-type *ABCA4* midigene condition (i), sequences of the correct transcript from mutant *ABCA4* midigene condition (ii), sequences of the PE transcript from mutant *ABCA4* midigene condition (iii), AON-corrected transcript in mutant *ABCA4* midigene condition (iv) and mutant transcript in AON-treated *ABCA4* midigene (v) are shown. **B)** Sequencing results in fibroblasts: sequences of the correct *ABCA4* transcript from control fibroblasts condition (i), sequences of the PE *ABCA4* transcript from patient-derived fibroblasts condition (ii), AON-corrected transcript from patient-derived fibroblasts condition (iii), mutant transcript from AON-treated patient-derived fibroblasts condition (iv), AON artifact (v), cycloheximide artifact (vi). **C)** Sequencing results in photoreceptor precursor cells: sequences of the correct *ABCA4* transcript from control PPC condition (i), sequences of the PE *ABCA4* transcript from patient-derived PPCs condition (ii), AON-corrected transcript in patient-derived PPCs condition (iii), mutant transcript in AON-treated in patient-derived PPCs condition (iv).

A. Sequencing results HEK-midigene system

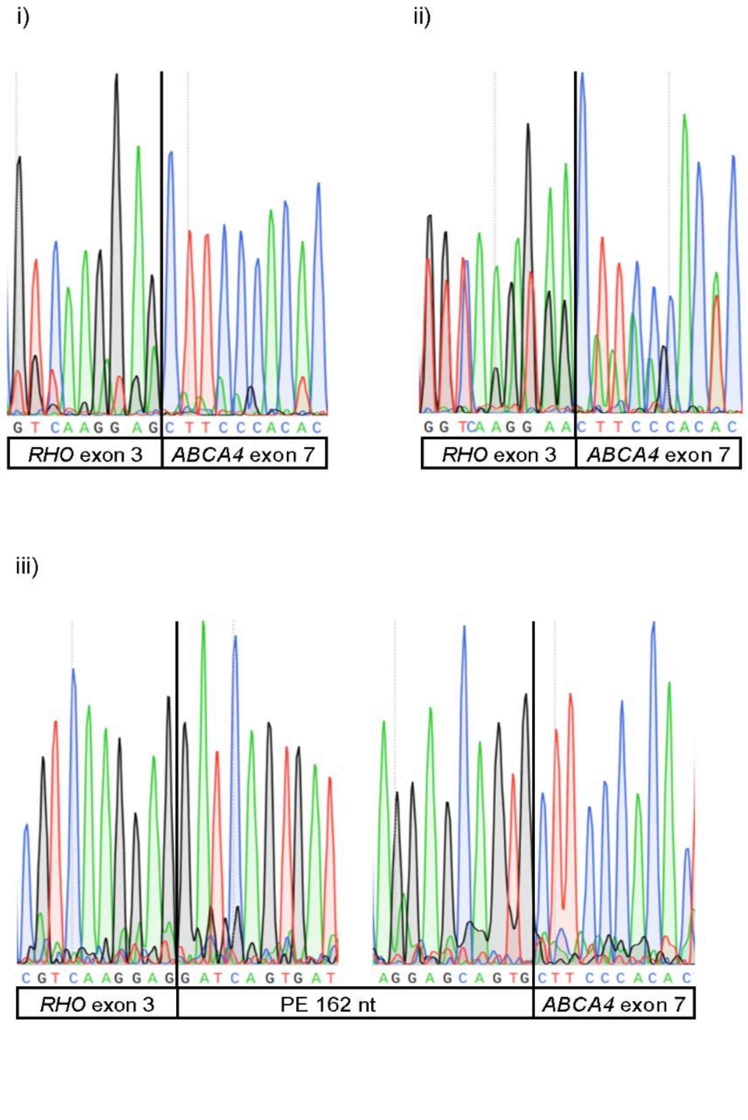

iv)

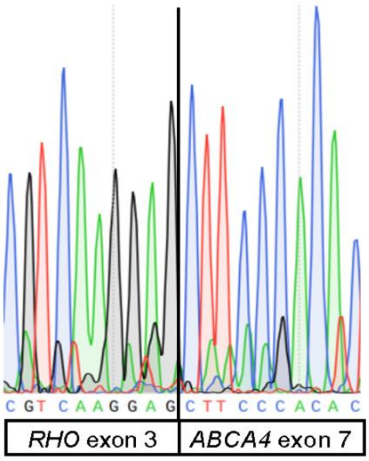
C G T C A A G G A G C T T C C C A C A C
RHO exon 3
ABCA4 exon 7

v)

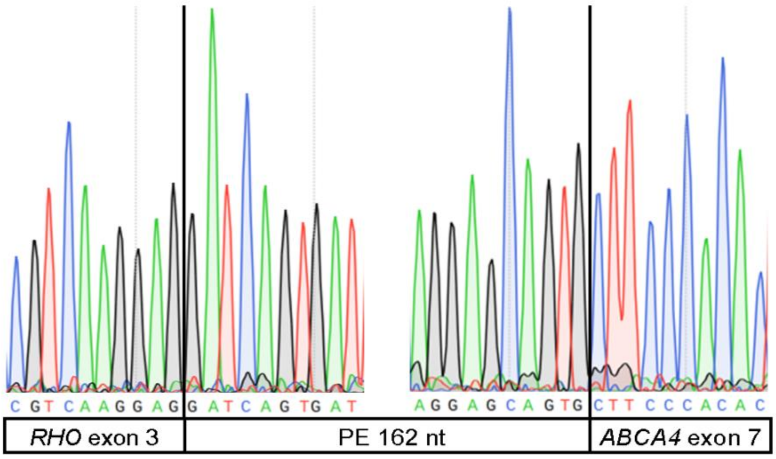
C G T C A A G G A G G A T C A G T G A T
A G G A G C A G T G C T T C C C A C A C
RHO exon 3
PE 162 nt
ABCA4 exon 7

B. Sequencing results fibroblasts

i)

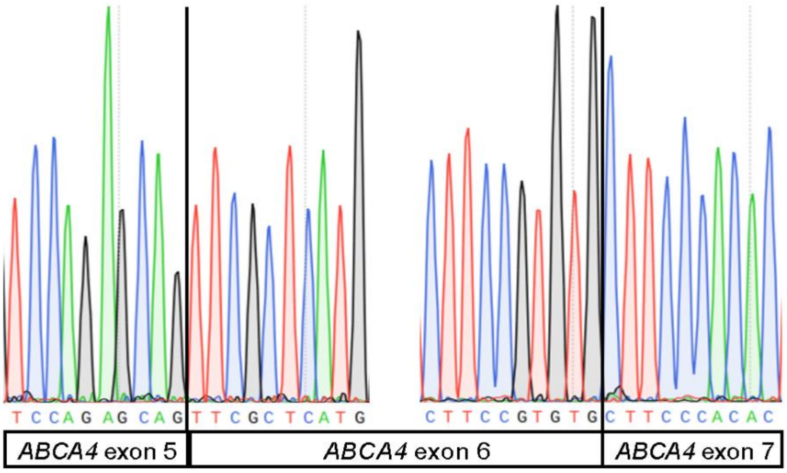

ii)

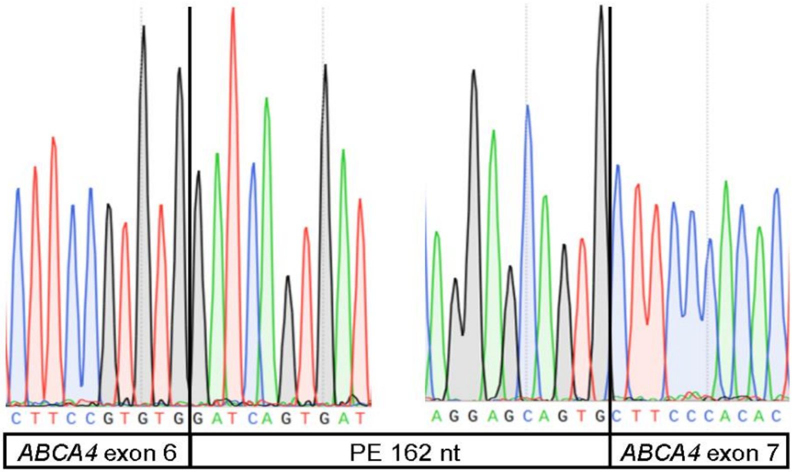

iii)

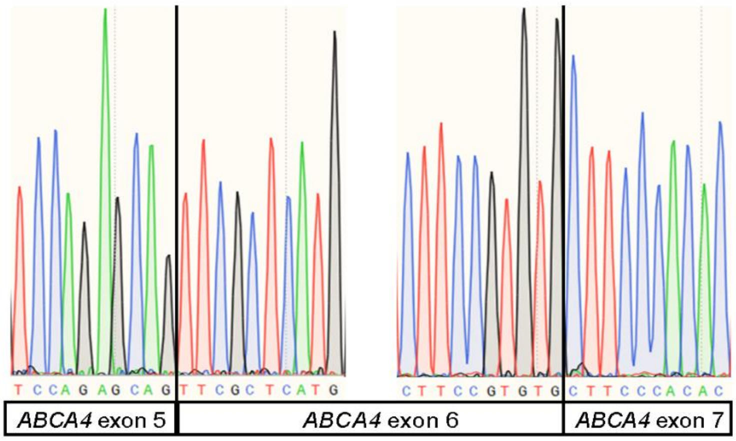
ABCA4 exon 5
ABCA4 exon 6
ABCA4 exon 7

iv)

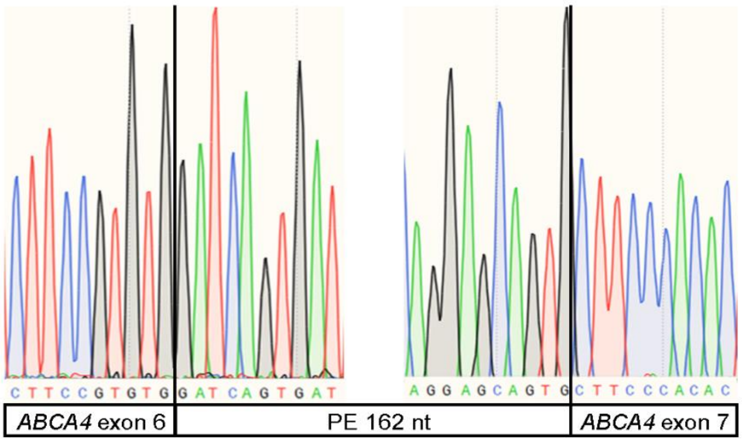
ABCA4 exon 6
PE 162 nt
ABCA4 exon 7

v)

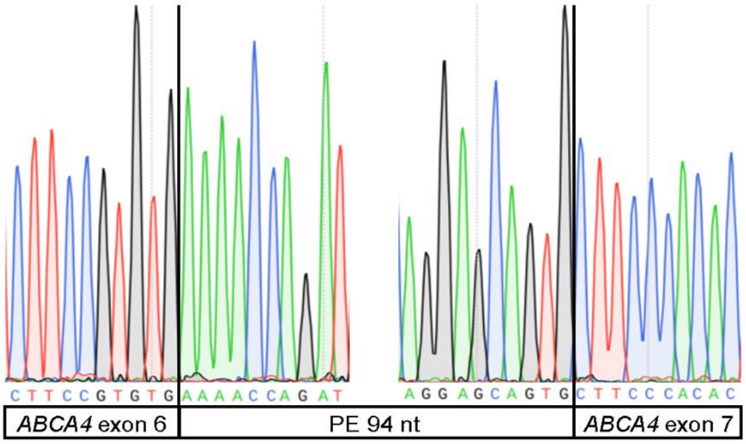
C T T C C G T G T G A A A A C C A G A T
A G G A G C A G T G C T T C C C A C A C
ABCA4 exon 6
PE 94 nt
ABCA4 exon 7

vi)

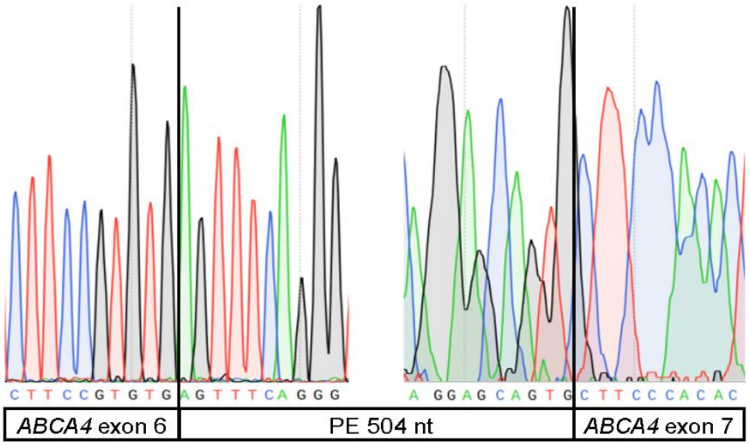
C T T C C G T G T G A G T T T C A G G G
A G G A G C A G T G C T T C C C A C A C
ABCA4 exon 6
PE 504 nt
ABCA4 exon 7

C. Sequencing results photoreceptor precursor cells

i)

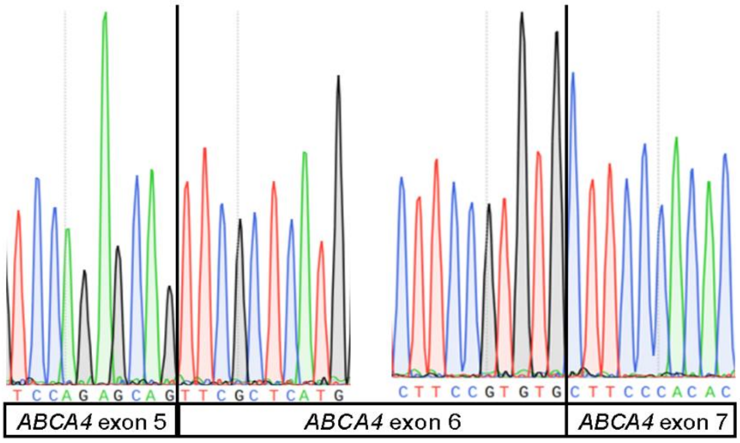

ii)

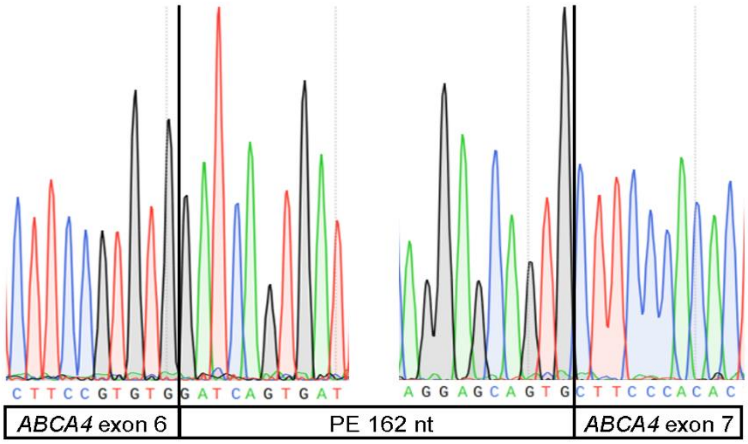

iii)

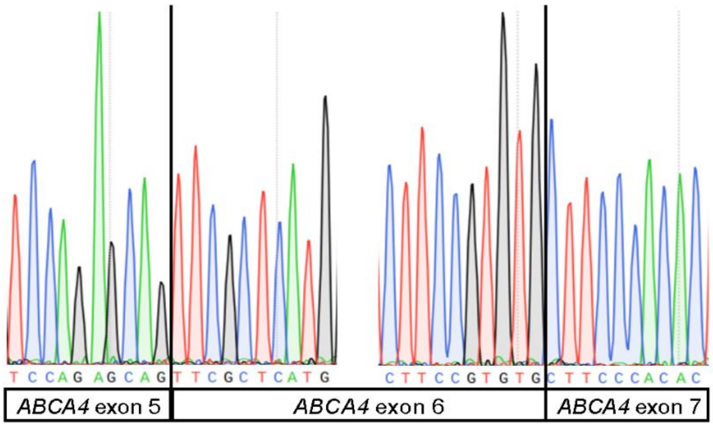

TCCAGAGCAGTTCGCTCATG
CTTCCGTGTGCTTCCCACAC
ABCA4 exon 5
ABCA4 exon 6
ABCA4 exon 7

iv)

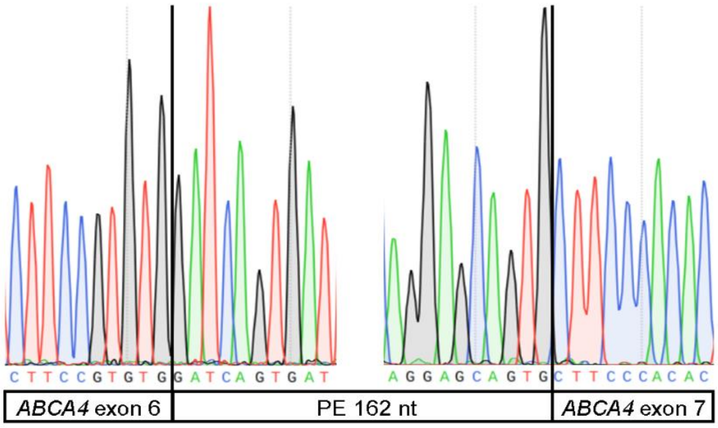

CTTCCGTGTGGATCAGTGAT
AGGAGCAGTGCTTCCCACAC
ABCA4 exon 6
PE 162 nt
ABCA4 exon 7

Supplementary Figure S2. AON-based rescue for the deep-intronic c.769-784C>T variant in patient-derived fibroblasts. The nine AONs and SON were tested in patient-derived fibroblasts. AONs and SON were transfected to patient-derived fibroblasts carrying the c.769-784C>T variant and were labelled from A1 to SON. Cycloheximide was used for cells labelled '+' and in all cells to which AONs and SON were transfected. The '-' indicates lack of cycloheximide treatment. The AON-not transfected control (CON) and patient-derived fibroblasts were used as a reference point for splicing-correction. MQ shows the negative control of the PCR reaction and amplification of exon 3 – exon 4 of *ACTB* gene was used as a loading control. **A)** In replicate 1 the PE band is stronger in the cycloheximide not treated control sample than in cycloheximide treated control. The PE band is weaker than expected in SON treated sample. AON-artifact is observed in sample treated with AON3. **B)** AON-artifact is observed in sample treated with AON1.

A. Antisense oligonucleotides screen in patient-derived fibroblasts, replicate 1

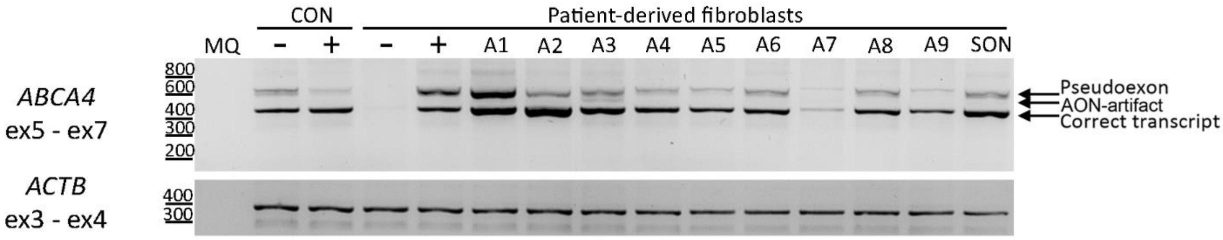

B. Antisense oligonucleotides screen in patient-derived fibroblasts, replicate 3

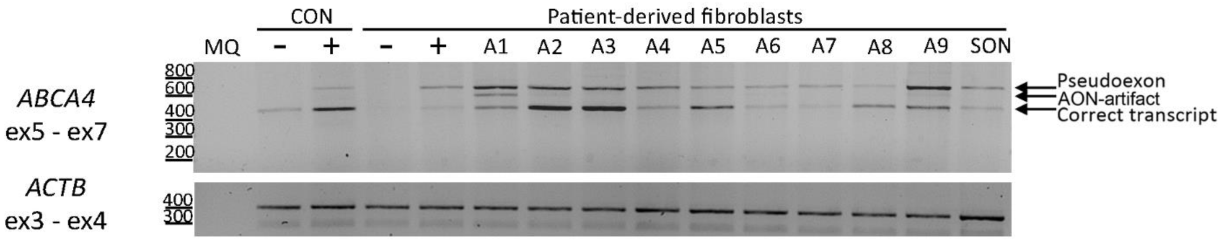

Supplementary Figure S3. AON-based rescue for the deep-intronic c.769-784C>T variant control-derived fibroblasts. A&B) Four AONs and SON were tested in control-derived fibroblasts. AONs and SON were transfected to control-derived fibroblasts and were labelled from A2, A5, A7, A9 and SON. Cycloheximide was used for cells labelled '+' and in all cells to which AONs and SON were transfected. The '-' indicates lack of cycloheximide treatment. The AON-not transfected control (CON) and patient-derived fibroblasts (PAT) were used as a reference point for splicing-correction. **C)** The graph represents the semi-quantification of the resulting RT-PCR products, showing the % of correct and pseudoexon (PE) inclusion transcript. Statistically significant PE exclusion was achieved for all four AONs. Milli-Q water (MQ) shows the negative control of the PCR reaction and amplification of exon 3 – exon 4 of *ACTB* gene was used as a loading control. Data (n=2) are presented as mean±SD and compared to the AON-untreated condition (*p<0.05, **p<0.01, ***p<0.001, ****p<0.0001).

A. Replicate 1 of AONs screen in control fibroblasts

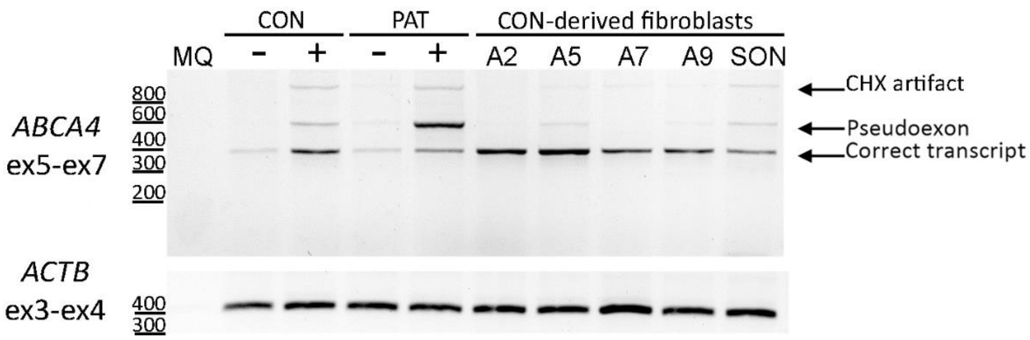

B. Replicate 2 of AONs screen in control fibroblasts

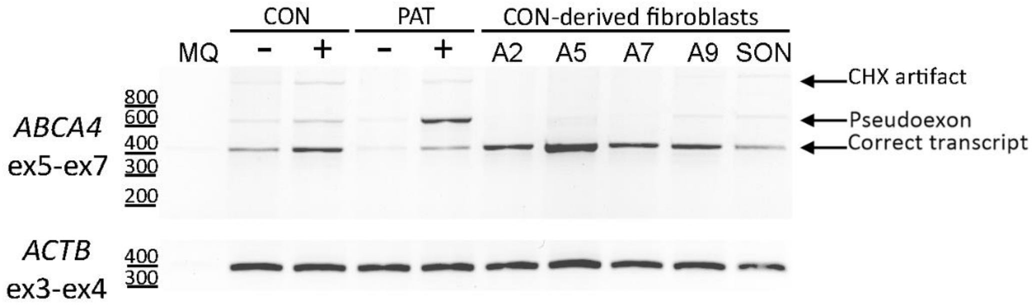

C. Average semi-quantification

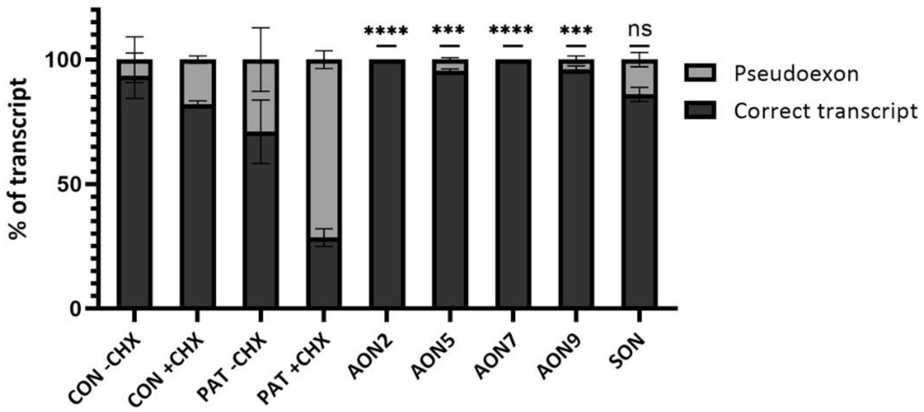

Supplementary Figure S4. Relative pluripotency and retinal gene expression profiles of photoreceptor precursor cells. Average gene expression profile of control iPSC differentiation for replicate 1 and 2. The abbreviations under each graph represents; wild-type induced pluripotent stem cells in black (WT iPS), wild-type photoreceptor precursor cells in grey (WT PPC), mutant carrying *ABCA4* c.769-784C>T variant in induced pluripotent stem cells in magenta (MT iPS) and mutant carrying *ABCA4* c.769-784C>T variant in photoreceptor precursor cells in orange (MT PPC) **(A).** Strong increase in expression was observed in early retina marker such as *PAX6* and with retina specific genes such as *ABCA4*. The expression of pluripotency markers *OCT3/4* and *OTX* were decreased as the cells acquired more retina like phenotype. The remaining retinal gene markers had variable expression. **B)** Average gene expression profile of patient-derived differentiation for replicate 1 and 2 showing the relative gene expression of differentiated PPCs relative to iPSC. An increase in expression was observed in early retina marker such as *PAX6* and with retina specific genes such as *ABCA4*. Other retinal markers were also increased such as *RCV1, RPE65* and *VMD2.* The expression of pluripotency markers *OCT3/4* and *OTX* were decreased as the cells acquired more retina like phenotype. The remaining retinal gene markers had variable expression.

A. Pluripotency and retinal gene expression in *ABCA4* wild-type rep1&2

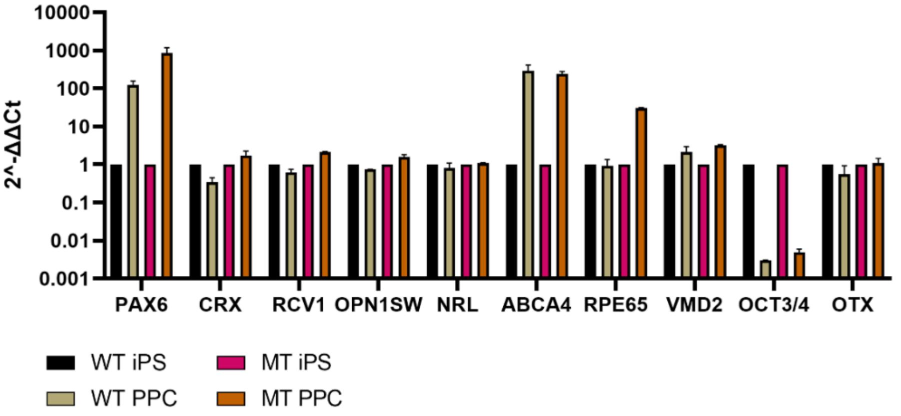

B. Pluripotency and retinal gene expression in *ABCA4* c.769-784C>T rep1&2

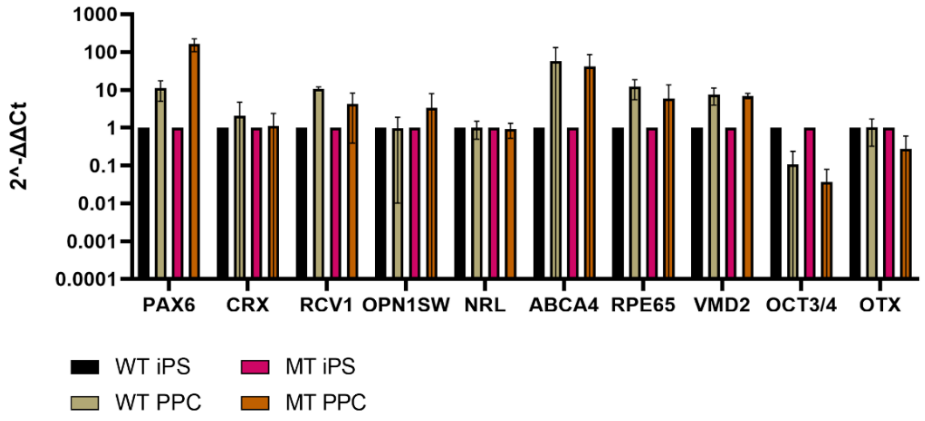

Supplementary Table S1. Primers for reverse transcription-PCR analysis and qPCR.

List of primers				
Forward Primer		Reverse Primer		Use
**Gene**	**Sequence 5' -> 3'**	**Gene**	**Sequence 5'-> 3'**	
*RHO*_exon3	CGGAGGTCAACAACGAGTCT	*ABCA4* exon7	CTTGAATTCTTGGTGACATATCAG	RT-PCR
*RHO*_exon5	ATCTGCTGCGGCAAGAAC	*RHO*_exon5	AGGTGTAGGGGATGGGAGAC	
ABCA4 exon5	GGAATACGAATAAGGGATATCTTG	*ABCA4* exon7	CTTGAATTCTTGGTGACATATCAG	
*ACTB*_exon3	ACTGGGACGACATGGAGAAG	*ACTB*_exon4	TCTCAGCTGTGGTGGTGAAG	
GUSB	AGAGTGGTGCTGAGGATTGG	*GUSB*	CCCTCATGCTCTAGCGTGTC	qPCR
ABCA4	CATCCTGTTCCACCACCTCA	*ABCA4*	CTGTGTCCTCCAACATGGCT	
CRX	CCCCAGTGTGGATCTGATG	*CRX*	CAAACAGTGCCTCCAGCTC	
NRL	GGCTCCACACCTTACAGCTC	*NRL*	AGCCAGTACAGCTCCTCCAG	
OCT3/4	GTTCTTCATTCACTAAGGAAGG	*OCT3/4*	CAAGAGCATCATTGAACTTCAC	
OPN1SW	TGGTATTGGCGTCTCCATC	*OPN1SW*	ACTCGCTGCGGTATTTGG	
OTX2	TATCTTAAGCAACCGCCTTACG	*OTX2*	GGAGGGGTGCAGCAAGTC	
PAX6	GCTGCAAAGAAATAGAACATCC	*PAX6*	TTGGCTGCTAGTCTTTCTCG	
RCV1	ACACCAAGTTCTCGGAGGAG	*RCV1*	ACTTGGCGTAGATGCTCTGG	
RPE65	TTACTACGCTTGCACAGAGACC	*RPE65*	GCCCCATTGACAGAGACATAG	
VMD2	TCAGTGTGGACACCTGTATGC	*VMD2*	AAGCTGTACACCGCCACAG	

Supplementary Table S2. Averaged semi-quantification analysis of transcripts from RT-PCR products from HEK293T-midigene screen, control and patient-derived fibroblasts screen and control and patient-derived PPC screen. The quantity of the different transcripts is represented as an averaged percent (%) of the total transcript for each condition or lane. SD: standard deviation.

	HEK293T-midigene MT screen																							
Condition	WT		MT		AON1		AON2		AON3		AON4		AON5		AON6		AON7		AON8		AON9		SON	
	%	SD	%	SD	%	SD	%	SD	%	SD	%	SD	%	SD	%	SD	%	SD	%	SD	%	SD	%	SD
Correct transcript	100%	0	79%	0.069	100%	0	100%	0	91%	0.053	90%	0.074	100%	0	100%	0	100%	0	93%	0.054	100%	0	73%	0.016
PE inclusion	0%	0	21%	0.069	0%	0	0%	0	9%	0.053	10%	0.074	0%	0	0%	0	0%	0	7%	0.054	0%	0	27%	0.016

	Patient-derived fibroblasts MT screen																											
Condition	CONT -CHX		CONT +CHX		PAT -CHX		PAT +CHX		AON1		AON2		AON3		AON4		AON5		AON6		AON7		AON8		AON9		SON	
	%	SD	%	SD	%	SD	%	SD	%	SD	%	SD	%	SD	%	SD	%	SD	%	SD	%	SD	%	SD	%	SD	%	SD
Correct transcript	88	0.203335	87	0.023401	100	0	35	0.114734	40	0.132073	78	0.125521	73	0.05148	51	0.228015	80	0.065079	51	0.167247	64	0.303136	77	0.053773	69	0.290877	48	0.212768
AON-artifact	0	0	0	0	0	0	0	0	9	0.107319	0	0	1	0.015389	1	0.014779	0	0	0	0	0	0	0	0	0	0	0	0
PE inclusion	12	0.203335	13	0.023401	0	0	65	0.114734	51	0.025342	22	0.125521	27	0.057593	48	0.223532	20	0.065079	49	0.167247	36	0.303136	23	0.053773	31	0.290877	52	0.212768

	Patient-derived fibroblasts WT screen																	
Condition	CONT -CHX		CONT +CHX		PAT -CHX		PAT +CHX		AON2		AON5		AON7		AON9		SON	
	%	SD	%	SD	%	SD	%	SD	%	SD	%	SD	%	SD	%	SD	%	SD
Correct transcript	93	0.093	81	0.004	43	0.298	27	0.038	100	0	95	0.001	100	0	96	0.023	87	0.016
PE inclusion	7	0.093	19	0.004	57	0.298	73	0.038	0	0	5	0.001	0	0	4	0.023	13	0.016

	Patient-derived PPC WT screen																					
Condition	CONT -CHX		CONT +CHX		PAT -CHX		PAT +CHX		AON2@0.5 µM		AON2@1 µM		AON5@0.5 µM		AON5@1 µM		AON7@0.5 µM		AON7@1 µM		SON 1@µM	
	%	SD	%	SD	%	SD	%	SD	%	SD	%	SD	%	SD	%	SD	%	SD	%	SD	%	SD
Correct transcript	100%	0	95%	0.013	85%	0.02	70%	0.003	97%	0.002	97%	0.015	97%	0.003	95%	0.006	97%	0.002	97%	0.004	96%	0.008
PE inclusion	0%	0	5%	0.013	15%	0.02	30%	0.003	3%	0.002	3%	0.015	3%	0.003	5%	0.006	3%	0.002	3%	0.004	4%	0.008

	Patient-derived PPC MT screen																					
Condition	CONT -CHX		CONT +CHX		PAT -CHX		PAT +CHX		AON2@0.5 µM		AON2@1 µM		AON5@0.5 µM		AON5@1 µM		AON7@0.5 µM		AON7@1 µM		SON 1@µM	
	%	SD	%	SD	%	SD	%	SD	%	SD	%	SD	%	SD	%	SD	%	SD	%	SD	%	SD
Correct transcript	100%	0	97%	0.015	84%	0.056	66%	0.058	95%	0.024	92%	0.034	79%	0.009	81%	0.077	95%	0.017	97%	0.005	74%	0.025
PE inclusion	0%	0	3%	0.015	16%	0.056	34%	0.058	5%	0.024	8%	0.034	21%	0.009	19%	0.077	5%	0.17	3%	0.005	26%	0.025
